# Phosphaturic Mesenchymal Tumor: An Unusual Cause of Stress Fractures Presenting to Sports Medicine

**DOI:** 10.7759/cureus.26847

**Published:** 2022-07-14

**Authors:** Rafat H Solaiman, Christian M Ogilvie

**Affiliations:** 1 Orthopedic Surgery, University of Minnesota Medical School, Minneapolis, USA

**Keywords:** sports medicine, oncology, orthopedic surgery, intermetatarsal, stress fractures, tumor-induced osteomalacia, phosphaturic mesenchymal tumor

## Abstract

Phosphaturic mesenchymal tumors (PMT) are a rare neoplasm oftentimes associated with tumor-induced osteomalacia (TIO). The non-specific presentation and symptoms of these pathologies make them difficult to diagnose. We report a case of a 52-year-old patient with an intermetatarsal phosphaturic mesenchymal tumor who presented to the orthopedic sports medicine clinic with metabolic deficiencies and bilateral subtrochanteric cortical stress fractures indicative of osteomalacia. The tumor was entirely resected within nine months of symptom onset and has shown no recurrence at the one-year follow-up. This case report characterizes an unusual cause of stress fractures presenting to orthopedic sports medicine clinics and the variability in the presentation of phosphaturic mesenchymal tumors.

## Introduction

Phosphaturic mesenchymal tumors (PMT) are a rare neoplasm associated with tumor-induced osteomalacia (TIO) [1]. Mechanistically, the tumor overproduces fibroblast growth factor 23 (FGF23), a phosphatonin, which inhibits proximal renal tubule reabsorption of phosphate and inhibits 1-alpha hydroxylase production. Consequently, circulating phosphate and 1,25-dihydroxyvitamin D are reduced, leading to renal phosphate wasting, improper bone mineralization, and osteomalacia [2,3]. Tumor-induced osteomalacia is a paraneoplastic syndrome that causes bone pain, fractures, muscle weakness, and is usually caused by mesenchymal tumors. Overproduction of FGF23 by the tumor causes several of these symptoms due to metabolic dysfunction [4]. Prompt surgical resection of PMTs is imperative for reversing metabolic dysfunction of bone.

The non-specific symptoms of TIO and associated PMTs make them difficult to diagnose. Retrospective studies have found the average time between symptom presentation and surgical tumor resection to be greater than five years [1,5]. Despite inducing osteomalacia, >97.5% of PMTs are benign, and complete tumor resection reverses TIO [6,7]. Less than 500 cases of PMTs have been reported in the literature [8]. Presentation of stress fractures and osteomalacia caused by a tumor to a sports medicine clinic is extremely unusual. However, a common early sign of underlying PMT is stress fractures, suggesting the possibility that these undiagnosed patients may be referred to sports medicine. We present a unique case of stress fractures and an underlying PMT presenting to the sports medicine clinic.

To help physicians who treat metabolic bone disease and stress fractures diagnose and treat this rare disease, we report a case of a non-malignant PMT between the second and third metatarsal of the left foot with TIO. The patient was informed that data concerning the case would be submitted for publication and provided his consent.

## Case presentation

**Figure 1:**
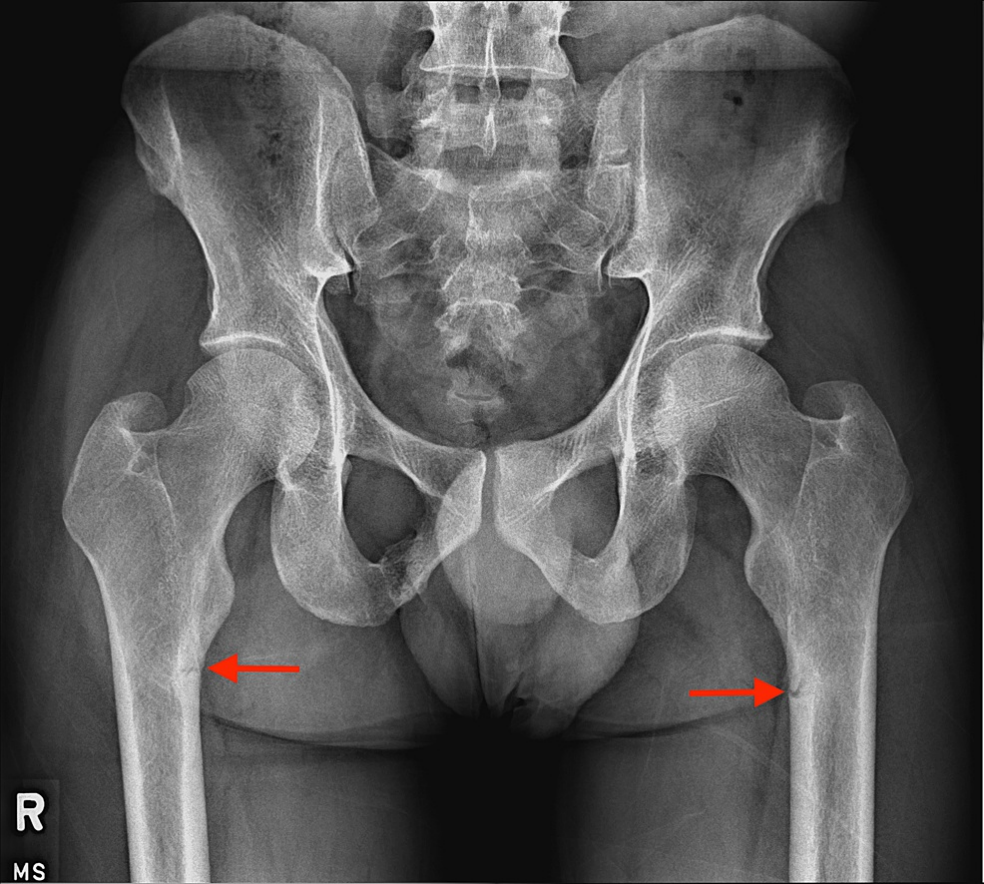
Radiograph of the pelvis and hip showing proximal femoral shaft lucencies indicative of stress fractures

A 52-year-old man presented to an outpatient clinic with intermittent, sharp pains upon hip flexion. He was seen by a physician’s assistant and referred to orthopedics sports medicine. He presented to the sports medicine physician with antalgic gait and abnormal internal rotation of the left hip but normal hip strength. An X-ray of the pelvis and hip showed proximal femoral shaft lucencies that were incomplete fractures indicative of osteomalacia (Figure 1). In a follow-up appointment two months later, a basic metabolic panel was ordered and revealed a low vitamin D level of 12 ug/L (normal range: 20-75 ug/L). After the lab results, he was put on 1000 mg/day of calcium and 2000 IU/day of vitamin D.

**Figure 2:**
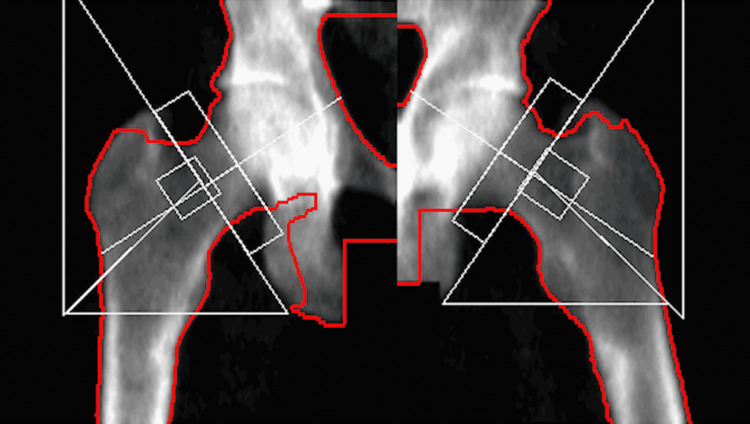
Bone density test of the hips indicating osteopenia in the right and left hip with respective T-scores of -2.0 and -2.1

At the one-month follow-up with the sports medicine physician, his pain had spread bilaterally to both hips and he was walking with a cane. The vitamin D supplementation returned levels to 36 ug/L but the radiographic scans showed no healing of the fractures. Four months later, the subtrochanteric cortical fractures remained bilaterally and a dual-energy X-ray absorptiometry (DEXA) scan was ordered. The DEXA scan revealed a T-score of -2.1 in the left hip, indicating an increase in fracture risk due to osteopenia (Figure 2).

**Figure 3:**
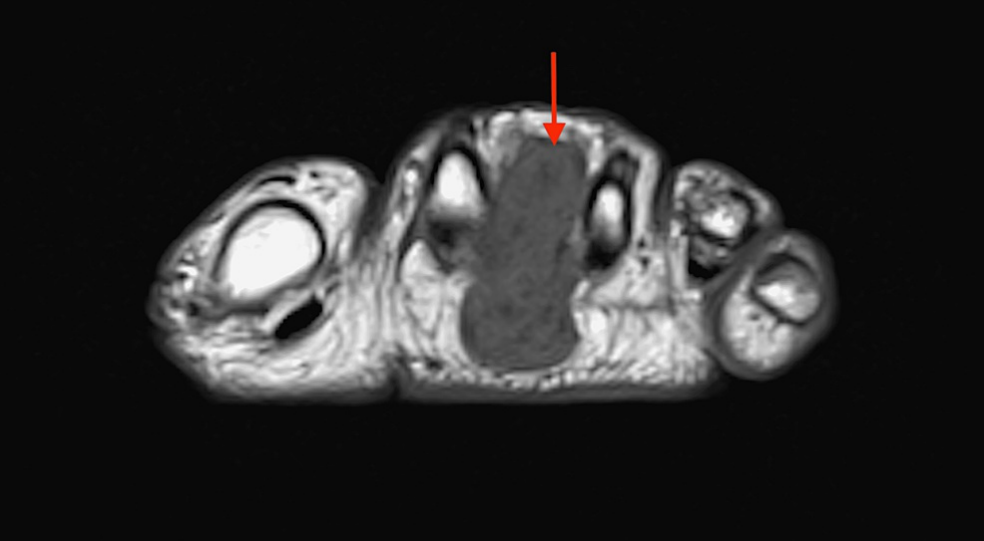
Magnetic resonance imaging displaying a soft-tissue mass with low signal intensity between the second and third metatarsal in a T-1 weighted sequence

**Figure 4:**
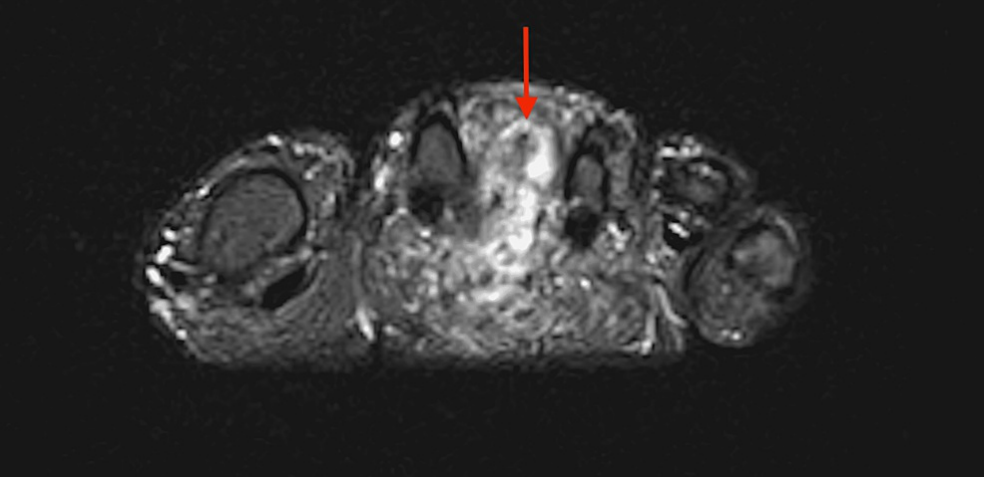
Magnetic resonance imaging displaying a soft-tissue mass with high signal intensity between the second and third metatarsal in a short tau inversion recovery (STIR) imaging sequence

Four days later, he presented to urgent care with left foot pain and swelling that became worse with weight-bearing. A soft-tissue mass was noted near his second metatarsal, and he was referred to see a podiatrist two weeks later. The patient noted that he noticed the intermetatarsal mass six months prior. The podiatrist ordered an MRI and was concerned that the mass could be a soft-tissue sarcoma (Figure 3 and Figure 4). The patient was referred to orthopedic oncology, and three days later he was seen in the orthopedic oncology clinic for a biopsy of the mass.

**Figure 5:**
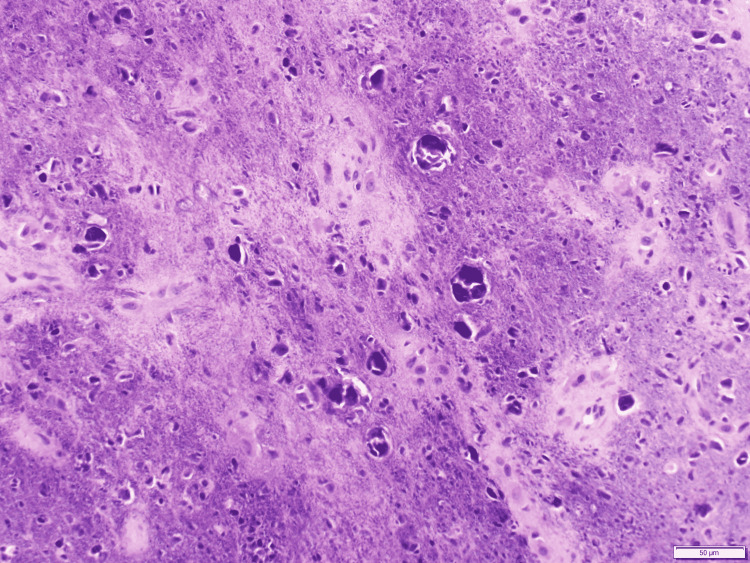
Histopathology from the tumor biopsy showing hypocellularity with myxoid matrix, grungy calcification, and bland nuclei

Histological examination of the mass demonstrated a hypocellular tumor with bland stellate and spindle cells embedded in a smudgy basophilic matrix (Figure 5). Occasional multinucleated giant cells were observed. Immunohistochemistry stains showed the lesional cells as positive for CD56 and erythroblast transformation-specific (ETS)-related gene (ERG), supporting the diagnosis of a PMT. Additional laboratory testing was ordered and revealed normal calcium level, normal vitamin D level, low phosphorus level, elevated alkaline phosphatase, and elevated FGF23 in serum (Table 1). Urine testing revealed an elevated phosphorous concentration. Marginal resection of the tumor was performed. The resected tumor measured 4.5x4.0x1.2cm. At the one-year follow-up postoperatively, the patient reported no sign of tumor regrowth and no new fractures. His hip pain resolved, and he was no longer using assistive devices to walk.

**Table 1:** Lab values supplementing our phosphaturic mesenchymal tumor diagnosis *Patient taking exogenous supplements FGF23: Fibroblast growth factor 23

Lab Test	Lab Value	Normal Range
Calcium*	9.8 mg/dL	8.5-10.2 mg/dL
Vitamin D*	43 ug/L	20-75 ug/L
Phosphorous	1.4 mg/dL	2.5-4.5 mg/dL
Alkaline Phosphatase	262 IU/L	40-150 IU/L
FGF23	363 RU/mL	<180 RU/mL

## Discussion

Phosphaturic mesenchymal tumors are an exceptionally rare disease with less than 500 cases ever reported [8]. Most PMTs occur in middle-aged patients, with 95% of tumors localized to the extremities and appendicular skeleton [9]. Phosphaturic mesenchymal tumors are almost always associated with TIO [1]. Common symptom presentation, similar to this case, includes gradual bone pain, myopathies, generalized weakness, and fractures. The non-specific elusive symptoms of PMTs make diagnosis difficult and oftentimes delayed. The average time from symptomatic onset to surgical resection of PMTs is greater than five years [1,5]. We present a case of an unusual cause of stress fractures in a patient presenting to a sports medicine clinic. The fractures were later found to be associated with an intermetatarsal PMT inducing osteomalacia. To the best of our knowledge, this is the first documentation of an underlying PMT presenting to a sports medicine clinic and is a valuable disease for sports medicine physicians to be aware of.

The diagnostic workup for PMTs requires a physical examination, imaging, laboratory testing, and histology. Symptom presentation of osteomalacia is common before the diagnosis of a PMT [10]. In this report, our patient presented with bone pain, antalgic gait, and abnormal hip rotation. The X-ray imaging revealed multiple incomplete subtrochanteric cortical fractures bilaterally, and laboratory testing identified a vitamin D deficiency. All these features are characteristic of osteomalacia and can be mitigated by vitamin D and calcium supplementation to correct for improper bone metabolism [11]. Stress fractures can be caused by a variety of conditions. More apparent causes may include trauma, osteoporosis, myositis ossificans, and soft-tissue calcifications, while infections and tumors can also be underlying causes [12]. Prolonged bisphosphonate use has also been associated with the risk of developing stress fractures [13]. Our patient had persistent non-healing fractures after vitamin D and calcium supplementation indicative of an additional underlying condition [14].

Laboratory testing is also a key indicator of a PMT. Serum FGF23 is commonly overexpressed by PMTs and inhibits both proximal renal tubule reabsorption of phosphate and production of 1-alpha hydroxylase leading to osteomalacia [2,3]. Decreased phosphate reabsorption causes increased excretion of phosphate in urine and decreased serum phosphorous. Likewise, decreased 1-alpha hydroxylase production causes vitamin D deficiency. Decreased serum phosphorous and increased FGF23 and alkaline phosphatase, as seen in our patient, are common lab abnormalities associated with TIO [8].

The common lack of a symptomatic soft-tissue mass in patients with TIO makes PMT difficult to diagnose. The acute pain caused by the intermetatarsal mass in our patient’s left foot at a relatively early stage of TIO symptom onset was unusual and allowed for a timely biopsy and diagnosis of PMT. If history and physical exam cannot identify a soft-tissue mass, different imaging modalities can be used to identify PMTs. When PMTs are not identified on physical examination, Gallium 68 (68Ga) 1,4,7,10-tetraazacyclododecane-1,4,7,10-tetraacetic acid (DOTA)-octreotate (DOTATATE, GaTate) positron emission tomography (PET)/computed tomography (CT), a newer modality to detect neuroendocrine tumors, can detect up to 100% of tumors [15]. However, this imaging modality is not always available at institutions and there remains no clear consensus on its utility over positron emission tomography with 2-deoxy-2-(fluorine-18)fluoro- D-glucose integrated with computed tomography (18F-FDG PET/CT), a more commonly available imaging modality [16]. An MRI can be utilized to further characterize the tumor after detection. The MRI characteristics of PMTs are non-specific, but the imaging modality is sensitive to detect masses. Small PMTs predominantly display a homogenous and intermediate or hyperintense signal on a T1-weighted image [16]. A T1-weighted MRI revealed an unusual hypointense and homogenous soft-tissue mass on our patient’s left foot (as seen above in Figure 3). A short tau inversion recovery sequence (STIR) can further help localize a PMT via MRI due to the high signal intensity of the tumor in these scans, which was characterized in our STIR sequence (as seen above in Figure 4).

A biopsy of the tumor in our patient showed characteristic histopathological and immunohistochemical features of a PMT. The PMT displayed bland, spindled, and stellate shape cells embedded in a smudgy basophilic matrix. Other common features seen in PMTs include cells with vesicular chromatin and a sheet-like arrangement [17]. The tumor also contained interspersed multinucleated giant cells, a less consistent feature among PMTs. Complete surgical resection of the tumor revealed a central hemorrhage characteristic in PMTs. Common immunohistochemical features found in the lesioned cells included CD56 and ERG. Additional positive markers include FGF23, fibroblast growth factor receptor 1 (FGFR1), B-cell lymphoma 2 (BCL-2), muscle-specific actin, and epithelial membrane antigen (EMA). However, variability is common in the markers present.

Due to the variability and rarity of PMTs, early diagnosis is oftentimes difficult. However, the majority of PMTs are benign, allowing marginal resection to be a curative treatment for TIO [7,18]. Increasing awareness among sports medicine physicians about this disease and adding to the body of literature in the variable clinical cases is crucial in decreasing the time gap between symptom onset and surgical resection of PMTs, and ensuring diagnoses aren’t missed. In cases of unusual stress fractures, asking patients about soft-tissue masses may help with early diagnosis, as this patient had an asymptomatic mass for months during his metabolic workup before the mass was biopsied. When osteomalacia is suspected, especially in cases without a clear underlying cause, we suggest adding FGF23, phosphorous, and alkaline phosphatase blood levels to laboratory testing. Detection of elevated FGF23 and hypophosphatemia are indicators of possible TIO and should prompt further diagnostic workup to identify the underlying tumor [19].

## Conclusions

Phosphaturic mesenchymal tumors and associated tumor-induced osteomalacia can be an unusual cause of stress fractures. Presentation of these tumors can be highly variable and may present to the sports medicine clinic. In cases of unusual stress fractures, patients should be asked about the presence of soft-tissue masses. When osteomalacia is suspected, especially in cases without a clear underlying cause, detection of elevated FGF23 and hypophosphatemia are indicators of possible tumor-induced osteomalacia and should prompt further diagnostic workup to identify an underlying tumor.
